# Gender Difference of the Relationship between Arterial Stiffness and Blood Pressure Variability in Participants in Prehypertension

**DOI:** 10.1155/2019/7457385

**Published:** 2019-06-25

**Authors:** Yang Lan, Huan Liu, Jinbo Liu, Hongwei Zhao, Hongyu Wang

**Affiliations:** Vascular Medicine Center, Peking University Shougang Hospital, Beijing, China

## Abstract

*Aim. *The association of pressure load with elasticity in vascular system has not been studied fully. We proposed a hypothesis whether gender could modify the association of blood pressure variability (BPV) and arterial stiffness assessed by carotid-femoral pulse wave velocity (CF-PWV) in prehypertensive patients.* Methods. *24h ambulatory blood pressure monitoring (24h-ABPM) and CF-PWV were measured in 723 participants with prehypertension. Univariate and multivariate regression analyses of these clinical and biological parameters were performed in total population, male and female.* Results. *A total of 723 participants (mean age 59.76 ± 12.37years, male 329 and female 394) were enrolled into the study. Compared with female, body mass index (BMI), fasting plasma glucose (FPG), uric acid (UA), and homocysteine (HCY) were significantly higher (all p < 0.05). Arterial stiffness (CF-PWV, male versus female, 10.89 ± 2.50 versus 10.33 ± 2.13 m/s, p=0.004) and BPVs (male versus female, 24 h SBPV 13.2 ± 5.11 versus 13.03 ± 5.20; 24 h DBPV 10.34 ± 3.87 versus 9.64 ± 3.59; N SBPV 11.90 ± 6.60 versus 10.94 ± 4.79; N DBPV 9.64 ± 5.87 versus 8.20 ± 4.48, all p<0.05) were higher in male. Multivariable linear regression analysis showed that 24 h BPV were linearly and positively related to CF-PWV in total population (24h SBPV, B=0.033; 24 h DBPV, B=0.035, both P<0.05) and female (24h SBPV, B=0.041; 24h DBPV, B=0.067, both P<0.05) independent of traditional risk factors and medications.* Conclusion. *BPV was independently associated with arterial stiffness in total population and the relation was modified by gender. 24 h BPVs in prehypertensive patients were useful to identify the early arterial stiffness.* Clinical Trials Registration*. This trial was registered with Clinical Trials.gov Identifier: NCT02569268.

## 1. Introduction

Prehypertension was defined as SBP of 120–139 mmHg and/or a DBP of 80–89 mmHg, and the prevalence of prehypertension was increased in the decades [1]. Ambulatory blood pressure monitor (ABPM) was secure and wearable medical devices and wildly used in clinical practice [2]. Blood pressure variability (BPV) increased with elevated average blood pressure and age [3] and was directly related to target organ damage, including left ventricular hypertrophy, coronary artery disease (CAD) and events, stroke, subclinical ischemic injury, kidney damage, and endovascular damage [4–6]. Previous study indicated that short-term variability of 24-hour SBP showed an independent and moderate relation to aortic stiffness in hypertension [7]. BPV, most widely used to assess the fluctuations of blood pressure, were standard deviation (SD) of both the systolic blood pressure (SBP) and the diastolic blood pressure (DBP) in 24-hour ambulatory BP recordings [8], although the SD did not reflect the steepness or rapidity in hypertensive individuals [9]. BPV could predict cardiovascular diseases [10, 11], and it had been proved as an additional and independent predictor of risk in young [12] and healthy population [13]. A meta-analysis showed that, for every 10-20 mmHg increased in SBP and DBP, the risk of cardiovascular disease and mortality was increased twofold, even in a normal levels of BP (115/75 mm Hg) [14].

Arterial stiffness was assessed by noninvasive measurement of carotid-femoral pulse wave velocity (CF-PWV), which had been recommended to assess the risk of future vascular events [15, 16]. The prevalence of arterial stiffness in subjects with prehypertension was unclear [17]. BPV reflected spontaneous fluctuations in blood pressure, and CF-PWV was a marker reflecting vascular function. Previous studies showed a steeper relationship between blood pressure variability and cardiovascular outcome, and the differences in physiological factors and risk factors in genders may affect the relationship between arterial stiffness and BPV [18, 19]. Therefore, we proposed the hypothesis that arterial stiffness was related to BPV and was modified by gender in participants with prehypertension.

## 2. Methods

### 2.1. Subjects

The participants of our study were selected from the part of Beijing Vascular Disease Patients Evaluation Study (BEST Study, Clinical Trials.gov Identifier: NCT02569268). BEST study was a post hoc analysis which recruited subjects aged 45-75 years through clinic or hospital from the western of Beijing, China. Our study recruited 723 subjects with prehypertension who completed 24-hour ambulatory blood pressure monitoring (24h-ABPM) and assessed arterial stiffness. Prehypertension was defined as SBP of 120–139 mmHg and/or a DBP of 80–89 mmHg [5], and we determined whether the subjects were in prehypertension according to the results of 24 h ABPM. Exclusion criteria were the presence of stroke, chronic heart failure, chronic renal failure, liver function impairment, systemic inflammatory diseases, infectious disease, autonomic dysfunction, cancer, and patients taking antihypertensive drugs. All subjects volunteered to participate in the study and provided the informed consent, which was based on the Declaration of Helsinki. The protocol of the study was proved by the ethic committee of Peking University Shougang Hospital.

### 2.2. Blood Pressure and Blood Pressure Variability (BPV) Measurement

Subjects were selected in the study underwent 24 h-ambulatory blood pressure monitoring and validated oscilloscope (ABPM 6100, Welch Allyn, Beijing, China) on a typical working day with appropriate exercise. The 24 h ABPM was programmed to automatically obtain BP recordings. Day and night were defined based on the waking and sleep time. The BP cuff was located on the participant's nondominant arm and the cuff size was determined by the upper arm circumference. 24 h ABPM parameters included 24-hour systolic blood pressure (24h SBP), 24-hour diastolic blood pressure (24h DBP), 24-hour pulse pressure (24h PP), 24-hour systolic blood pressure standard deviation (24h SBP SD), 24-hour diastolic blood pressure standard deviation (24h DBP SD), daytime systolic pressure (D SBP), daytime diastolic pressure (D DBP), daytime pulse pressure (D PP), nighttime systolic pressure (N SBP), nighttime diastolic pressure (N DBP), and night pulse pressure (D PP). We used standard deviation (SD) as the indicator of BPV.

### 2.3. Carotid-Femoral Pulse Wave Velocity (CF-PWV) Measurement

CF-PWV was simultaneously and automatically measured by the device Complior SP (Artech Medical, Pantin, France). After subjects rested in the supine position for 5-10 minutes, we preformed the measurement to obtain CF-PWV values. CF-PWV was primarily obtained by measuring the distance of two cuffs (carotid artery and femoral artery) and the conduction time of pulse wave. We used a correction factor of 0.8 to explain the difference between the measured distance between both cuffs and the reference distance [20]. The mean CF-PWV of three measurements was used for analysis.

### 2.4. Statistical Analysis

In the cross-sectional study, all continuous variables were expressed as mean (SD) or quartile ranges by gender. We used independent-samples Student's test to analyze the clinical features, 24 h ABPM recordings, and CF-PWV, and the results were presented by gender. P < 0.05 was considered to be of statistical significance. Multivariable linear regression was used to assess the relationship between CF-PWV and 24 h ABPM recordings in the total population, male, and female separately after adjusted for age (continuous), body mass index (BMI, kg/m2), heart rate (HR), smoking, family history, fasting plasma glucose (FPG), total cholesterol (TC), triglyceride (TG), high-density lipoprotein cholesterol (HDL-C), low-density lipoprotein cholesterol (LDL-C), high-sensitivity C-reactive protein (hs-CRP), diabetes, coronary artery disease (CAD), peripheral arterial disease (PAD), agents of hyperlipidemia, and agents of hypoglycemic. CF-PWV was used as the dependent variable, 24 h ABPM parameters; age and heart rate (HR) were independent variables. Statistical analysis was performed by SPSS 24.0 statistical software.

## 3. Results

### 3.1. General Clinical Features in Total, Male, and Female Population

The clinical features of the study population by gender were shown in Table 1. We recruited 723 participants, including 329 males (45.50%) and 394 females (54.50%) with mean age of 59.76 ± 12.37 years and BMI of 24.59 ± 3.66 Kg/m^2^. Compared with female, age, BMI, FPG, UA, and HCY levels were significantly higher in male (all p<0.05). Conversely, TC, HDL-c, and LDL-c were significantly higher in female.

### 3.2. 24h ABPM Recordings and Vascular Parameters according to Gender and Total Population

The summary statistics of 24 h ABPM recordings were shown in Table 2. The mean SBP (including 24 h SBP, D SBP, and N SBP) were higher in male, and 3-4mmHg higher than that in female. BPVs (including 24 h SBP SD, 24 h DBP SD, N SBP SD, and N DBP SD) were also significantly higher in male (all p < 0.05). Conversely, PP (including 24 h PP, D PP and N PP) were slightly higher in female (all p>0.1). CF-PWV, reflecting arterial wall stiffness, was also significantly higher in male (male versus female: 10.89 ± 2.50 versus 10.33 ± 2.13, p = 0.026). The records with history of vascular-related diseases and medications were shown in Table 3. In the present population, 152 (21.02%) subjects were in smoking status, and 93 (12.89%) were diabetes, 117 (16.32%) were coronary artery disease, 53 (7.33%) were peripheral arterial disease, and 519 (71.78%) were without vascular-related diseases.

### 3.3. The Results of Multivariable-Adjusted Linear Regression between 24h ABPMs and CF-PWV

**Figure 1:**
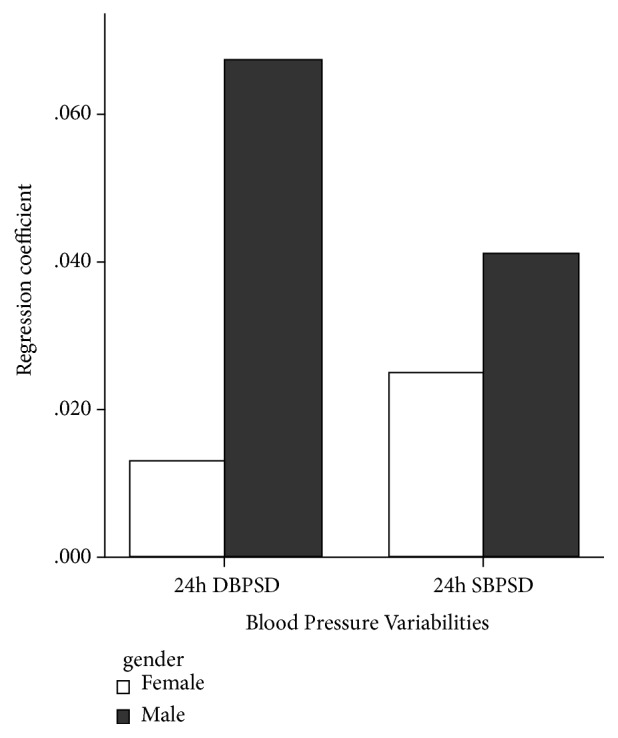
Comparison of linear regression coefficients between CF-PWV and BPVs in male and female.

Our researchers further analyzed the relationship between CF-PWV and BPV. In total population, CF-PWV was positively and linearly correlated with 24 h SBP SD and 24 h DBP SD (24h SBP SD, B=0.033, R^2^=0.368, P=0.003; 24 h DBP SD, B=0.035, R^2^=0.364, P =0.06, Table 4) independent of traditional risk factors and medications. And then we performed the analysis of the interaction between gender, BP, and BPV; it showed that gender difference may affect the relationship between 24 h SBP SD, 24 h DBP SD, and CF-PWV (see Table 5). In female, it found that arterial stiffness had the strongest linear relationship with 24 h SBP SD (B=0.041, R^2^=0.379, P=0.008) and 24 h DBP SD(B = 0.067, R^2^=0.379, P = 0.015). But the relationship did not occur in male (24h SBP SD, B=0.025, R^2^= 0.371, P=0.145; 24 h DBP SD, B=0.013, R^2^=0.367, P=0.633). We analyzed the difference of regression coefficients in male and female (see Figure 1). CF-PWV had obvious linear relationship with BPVs in female. In addition, the linear relationship between PP, age, and arterial stiffness was stronger in female (see Table 6). All the results were analyzed adjusting for age, gender, BMI, HR, SBP, DBP, family history, smoking status, FPG, TC, TG, HDL, LDL, HCY, hs-CRP, UA, CAD, PAD, diabetes, hypoglycemic drugs, and lipid-lowering drugs.

## 4. Discussion

In the hospital-based cross-sectional study of subjects in prehypertension, we determined the hypothesis that arterial stiffness was related to BPV and was also modified by gender. The results showed that BPVs and mean BP were different between male and female and significantly higher in male. CF-PWV was positively and linearly correlated with 24 h SBP SD, 24 h DBP SD, PP, age, and HR in the total population. In female, BPVs (including 24 h SBP SD, 24 h DBP) were independent factors and linearly correlated with CF-PWV, but not in male. Women showed a steeper relationship between CF-PWV and age, PP.

Many studies consistently believed that BPV was associated with cardiovascular disease and cerebrovascular diseases. However, the BP level in patients with hypertension as a confounding factor may affect the relationship between BPV and cardiovascular disease. Previous study had showed that hypertension could increase the arterial stiffness and damage the baroreflex sensitivity, which in turn influenced BPV [21]. Therefore, we analyzed the relationship between BPV and arterial stiffness in participants with prehypertension. Our findings were also supported by some cross-sectional studies and longitudinal studies. Part of studies suggested that BPV was associated with carotid atherosclerosis [22], stroke [23, 24], and vascular events [25]. A 10-year follow-up study showed that higher long-term SBP variability may be a risk factor for arterial stiffness progression independent of mean BP, as a result of that, higher systolic blood pressure (SBP) variability may reduce bioavailability of nitric oxide and increase vascular smooth muscle cell proliferation [26]. A recent study reported that per SD increased in systolic BPV was statistically significantly associated with 0.10 m/s (95% CI, 0.01–0.20) increased in CF-PWV, greater carotid circumferential wall tension, and greater intima-media thickness [24]. Some studies also suggested that BPV was associated with target organ damage in hypertension [4, 27]. However, it reported that the relationship between BPV and cardiovascular events was not consistent. A research (almost 3,000 participants in the Flemish population study) failed to manifest any association between visit-to-visit BPV and cardiovascular outcomes [28].

In addition, we found a gender difference in the relationship between BPV and arterial stiffness. We performed the analysis of the interaction between gender, BP, and BPV; it showed that gender difference may affect the relationship between 24 h SBP SD, 24 h DBP SD, and CF-PWV. After adjusting for traditional risk factors and medications, BPV and CF-PWV showed an independent and positive correlation in female, and the linear regression coefficient was significantly higher. There were only a few studies focused on gender in the reports of BPV and CF-PWV, and the conclusions were also inconsistent. A study showed that BPV was an independent predictor of cardiovascular events in women [28]. And a United Kingdom study of more than 20,000 large-scale population studies with a follow-up of 29 years showed a brief higher reading of diastolic blood pressure (indicating higher blood pressure variability), cardiovascular disease, and all-cause mortality were closely related only to women, not men [19]. However, a recent study showed that 24 h SBPV was an independent factor in assessing carotid IMT in male, but not in female [22]. The pathophysiologic mechanisms were poorly defined, although many hypotheses were proposed with the hormonal component being the prevailing [29]. Studies showed that estrogen exerted a variety of beneficial cardiovascular effects and elevated estrogen levels in premenopausal women induced vasodilation, which in turn reduced aortic stiffness by acting on endothelial cells and smooth muscle cells. Decreased estrogen levels caused by ovarian function declining could diminish the beneficial effect, which ultimately contributed to an increase in cardiovascular risk [30]. Reports from the multiethnic study of atherosclerosis showed that hypertension events were positively correlated with testosterone and estradiol levels and negatively correlated with sex hormone binding globulin levels [31]. Therefore, we speculated that the gender differences in our findings may be caused by hormone levels. There may be gender difference in the impact of risk factors over time with differing critical periods or levels of impact [19].

Age contributed to the development of arterial stiffness [32]. It proved that arterial stiffness also exist in adolescents independent of blood pressure levels and further developed with time [33, 34]. Our results showed that the linear relationship between age, PP, and CF-PWV was significantly steeper in female. Elevated PP also was the result of arterial stiffness, especially in postmenopausal women [35, 36]. In a large-scale cohort study, it found that elevated PP was independently associated with cardiovascular and all-cause mortality, and the association was significantly steeper in female [37].

In the present study, further intense clinical research was needed to clarify the potential gender difference in hypertension and antihypertensive therapy, not only in the general population but also in specific subgroups, such as patients with diabetes, cardiovascular disease, and chronic kidney disease. Multicenter and large-scale sample are further needed to validate the results. Moreover, further basic research was of paramount importance to uncover the biological plausibility and the mechanisms which mediated the potential gender difference in hypertension and cardiovascular disease.

It is the first time to present an interesting clinical phenomenon that arterial stiffness was closely related to BPVs in the female population and independent of traditional risk factors. Our clinical phenomenon provided the basis that gender difference may affect vascular function and further promote the multicenter research about the relationship between BP, BPV, and vascular function. In addition, subjects were both from clinics and hospital to reduce sample selection bias. Also, some limitations existed. First of all, when assessing the variability of noninvasive ambulatory blood pressure monitoring techniques, the time frequency of blood pressure measurements was important and the results of the study should be confirmed by shorter interval dynamic blood pressure measurements. Second, we did not use weighted 24 h BP SD and the assessment of 24 h BPVs was difficult to replicate. Finally, our cross-sectional study did not adequately explain the causal relationship between 24-hour ABPM and arteriosclerosis and the relationship between BPV, CF-PWV, and vascular-related diseases.

## 5. Conclusion

All 24 h ABPM parameters were higher in male, except for PP. Blood pressure variability (BPV), age, pulse pressure, and heart rate were linearly positively correlated with CF-PWV, especially in female. The results indicated that stable blood pressure control may delay the development of atherosclerosis.

## Figures and Tables

**Table 1:** Clinical features of subjects in general, male and female population.

	Total (n=723)	Male (n=329)	Female(n=394)	p
Age (years)	59.76 ± 12.37	60.05 ± 13.87	59.52 ± 10.975	0.565
BMI (kg/m2)	24.59 ± 3.70	25.13 ± 3.68	24.15 ± 3.66	0.001^*∗∗*^
HR (beats/min)	68.68 ± 13.42	69.25 ± 14.56	68.20 ± 12.39	0.2941
FPG (mmol/L)	5.78 ± 1.45	5.95 ± 1.72	5.64 ± 1.15	0.011*∗*
UA (umol/L)	311.05 ± 80.54	347.80 ± 80.77	279.65 ± 65.81	0.001*∗∗*
TC (mmol/L)	4.86 ± 1.08	4.60 ± 1.06	5.09 ± 1.04	0.001^*∗∗*^
TG (mmol/L)	1.74 ± 1.48	1.85 ± 1.54	1.65 ± 1.32	0.115
HDL-c (mmol/L)	1.26 ± 0.52	1.13 ± 0.26	1.37 ± 0.65	0.001*∗∗*
LDL-c (mmol/L)	2.84 ± 0.86	2.81 ± 0.83	3.06 ± 0.83	0.001*∗∗*
Hs-CRP (mg/L)	1.27	1.28	1.27	0.754
	(0.64-2.95)	(0.61-3.17)	(0.61-2.62)	
HCY (umol/L)	13.92 ± 7.31	16.78 ± 8.33	11.36 ± 5.02	0.001^*∗∗*^

Values are expressed as mean ± SD for continuous variables and median for categorical variables (interquartiles). *∗* indicated p<0.05; *∗∗* indicated p<0.001.

BMI, body mass index; HR, heart rate; FPG, fasting plasma glucose; UA, serum uric acid; TC, total cholesterol; TG, triglyceride; HDL-C, high density lipoprotein cholesterol; LDL-C, low density lipoprotein cholesterol; hs-CRP, high sensitive C reactive protein; HCY, homocysteine.

**Table 2:** 24h ambulatory blood pressure monitoring parameters and CF-PWV of subjects in general, male, and female population.

	Total (n=723)	Male (n=329)	Female (n=394)	p
24-h ABPMS				

24h SBP	120.53 ± 13.84	122.57 ± 13.78	118.82 ± 13.69	0.001^*∗∗*^
24h DBP	70.77 ± 10.28	73.15 ± 10.66	68.78 ± 9.51	0.001^*∗∗*^
24h SBP SD	13.26 ± 5.62	14.12 ± 5.11	13.03 ± 5.20	0.043^*∗*^
24h DBP SD	9.96 ± 3.73	10.34 ± 3.87	9.64 ± 3.59	0.001^*∗∗*^
24h PP	49.74 ± 9.16	49.39 ± 9.23	50.03 ± 9.06	0.355

*Daytime ABPMS*				

D SBP	121.84 ± 14.30	123.88 ± 14.41	120.13 ± 13.99	0.001^*∗∗*^
D DBP	71.97 ± 10.96	74.41 ± 11.65	69.93 ± 9.92	0.001^*∗∗*^
D PP	50.03 ± 9.39	49.74 ± 9.59	50.27 ± 9.23	0.453

*Nighttime ABPMS*				

N SBP	115.64 ± 14.66	117.75 ± 14.74	113.89 ± 14.39	0.001^*∗∗*^
N DBP	66.88 ± 10.25	69.33 ± 10.75	64.84 ± 9.33	0.001^*∗∗*^
N SBP SD	11.37 ± 5.70	11.90 ± 6.60	10.94 ± 4.79	0.026^*∗*^
N DBP SD	8.85 ± 5.20	9.64 ± 5.87	8.20 ± 4.48	0.001^*∗∗*^
N PP	48.91 ± 10.21	48.42 ± 10.48	48.96 ± 9.70	0.478

*Arterial stiffness*				

CF-PWV	10.63 ± 2.39	10.89 ± 2.50	10.33 ± 2.13	0.004*∗*

Values are expressed as mean ± SD. *∗* indicated p<0.05; *∗∗* indicated p<0.001. CF-PWV, carotid femoral pulse wave velocity; 24 h SBP, 24-hour systolic blood pressure; 24 h DBP, 24-hour diastolic blood pressure; 24 h PP, 24-hour pulse pressure; 24 h SBP SD, 24-hour systolic blood pressure standard deviation; 24 h DBP SD, 24-hour diastolic Standard deviation of pressure; D SBP, daytime systolic pressure; D DBP, daytime diastolic pressure; D PP, daytime pulse pressure; N SBP, nighttime systolic pressure; N DBP, nighttime diastolic pressure; D PP, night pulse pressure.

**Table 3:** History of vascular related diseases and medications in total population.

Valuables	Records
Smoking status, no (%)	152 (21.02)
Family history, no (%)	180 (24.90)
Hypolipidemic drug, no (%)	160 (22.13)
Hypoglycemic drug, no (%)	54 (7.47)
Nitrate drug, no (%)	22 (3.04)
CAD, no (%)	117 (16.32)
PAD, no (%)	53 (7.33)
DM, no (%)	93 (12.86)
Without vascular-related diseases, no (%)	519 (71.78)

Vascular-related diseases were defined as CAD, stroke, PAD, hypertension, and DM, hyperlipidemia

**Table 4:** Association of CF-PWV with BPVs and PP in overall population, by multivariable-adjusted linear regression analysis.

	Total (n=723)		
	B	R^2^	p
24h SBP SD	0.033	0.368	0.003*∗*
24h DBP SD	0.035	0.364	0.060*∗*
N SBP SD	0.012	0.361	0.360
N DBP SD	0.006	0.362	0.672
24h PP	0.038	0.370	0.001*∗∗*
D PP	0.029	0.366	0.010*∗∗*
N PP	0.040	0.376	0.001*∗∗*
Age	0.084	0.368	0.001*∗∗*
HR	0.039	0.390	0.001*∗∗*

*∗* indicated p<0.05; *∗∗* indicated p<0.001.

Adjusted for age, gender, HR, BMI family-history, smoke, SBP, DBP, CAD, PAD, diabetes, UA, FPG, TG, TC, LDL, HDL, HCY, Hs-CRP, hypolipidemic drug, hypoglycemic drug, and nitrate drug.

**Table 5:** A term of interaction between BPV, BP and gender in the multivariate–adjusted linear regression model for total population.

Total (n=723)		
Variables	F	P for interaction
Gender*∗* 24 h SBP SD	7.478	0.001*∗∗*
Gender*∗* 24 h DBP SD	3.178	0.042*∗*
Gender*∗* N SBP SD	0.078	0.925
Gender*∗* N SBP SD	0.522	0.594
24h SBP SD *∗* SBP	0.558	0.456
24h DBP SD *∗* DBP	2.968	0.056
N SBP SD *∗* SBP	15.433	0.001*∗∗*
N SBP SD *∗* SBP	4.850	0.028

*∗* indicated p<0.05; *∗∗* indicated p<0.001.

**Table 6:** Association of CF-PWV with BPVs and PP in male and females, by multivariable-adjusted linear regression analysis.

	Male (n=329)			Female (n=394)		
	B	R^2^	P	B	R^2^	P
24h SBP SD	0.025	0.371	0.145	0.041	0.379	0.008*∗*
24h DBP SD	0.013	0.367	0.633	0.067	0.379	0.015*∗*
N SBP SD	0.005	0.362	0.796	0.023	0.376	0.206
N DBP SD	0.001	0.364	0.622	0.030	0.378	0.121
24h PP	0.013	0.364	0.470	0.061	0.398	0.001*∗*
D PP	0.004	0.363	0.790	0.052	0.391	0.001*∗*
N PP	0.018	0.364	0.240	0.060	0.412	0.001*∗∗*
Age	0.076	0.363	0.001*∗∗*	0.084	0.366	0.001*∗∗*
HR	0.031	0.363	0.001*∗∗*	0.020	0.366	0.047*∗*

*∗* indicated p<0.05; *∗∗* indicated p<0.001.

Adjusted for age, HR, BMI family-history, smoke, SBP, DBP, CAD, PAD, diabetes, UA, FPG, TG, TC, LDL, HDL, HCY, Hs-CRP, hypolipidemic drug, hypoglycemic drug, and nitrate drug.

## Data Availability

The data used to support the findings of this study are available from the corresponding author upon request.
